# Impact of Key Assumptions About the Population Biology of Soil-Transmitted Helminths on the Sustainable Control of Morbidity

**DOI:** 10.1093/cid/ciab195

**Published:** 2021-06-14

**Authors:** Carolin Vegvari, Federica Giardina, Veronica Malizia, Sake J de Vlas, Luc E Coffeng, Roy M Anderson

**Affiliations:** 1 London Centre for Neglected Tropical Disease Research, Department of Infectious Disease Epidemiology, Imperial College London, London, United Kingdom; 2 Medical Research Council Centre for Global Infectious Disease Analysis, Department of Infectious Disease Epidemiology, School of Public Health, Imperial College London, London, United Kingdom; 3 Department of Infectious Disease Epidemiology, School of Public Health, Imperial College London, London, United Kingdom; 4 Department of Public Health, Erasmus MC, University Medical Center Rotterdam, Rotterdam, The Netherlands; 5 The DeWorm3 Project, Natural History Museum, London, United Kingdom

**Keywords:** soil-transmitted helminthes, morbidity control, programme planning, mathematical modeling

## Abstract

The design and evaluation of control programs for soil-transmitted helminths (STHs) is based on surveillance data recording measurements of egg counts in the stool of infected individuals, which underpin estimates of the prevalence and average intensity of infection. There is considerable uncertainty around these measurements and their interpretation. The uncertainty is composed of several sources of measurement error and the limit of detection of fecal smear tests on the one hand, and key assumptions on STH biology on the other hand, including assumptions on the aggregation of worms within hosts and on the impact of density-dependent influences on worm reproduction. Using 2 independently developed models of STH transmission we show how different aspects of STH biology and human behavior impact on STH surveillance and control programs and how accounting for uncertainty can help to develop optimal and sustainable control strategies to meet the World Health Organization (WHO) morbidity target for STHs.

Soil-transmitted helminths (STHs) are a group of parasites that affect an estimated 1.5 billion people world-wide, predominantly in low-income settings with lack of access to basic hygiene and sanitation facilities [1]. The main STH species are roundworm (*Ascaris lumbricoides*), hookworm (*Necator americanus*, *Ancylostoma duodenale*), and whipworm (*Trichuris trichiura*). STH infections cause considerable morbidity in vulnerable populations and are associated with malnutrition, anemia, adverse pregnancy outcomes, impaired cognitive and physical development in children and educational underachievement [2, 3]. In 2001, the World Health Assembly passed a resolution to reduce STH-associated morbidity with the help of systematic STH control programs [4]. The main pillars of STH control programs are improved water, sanitation, and hygiene (WASH) facilities and preventive chemotherapy (PC) targeted at preschool-age children (pSAC), school-age children (SAC), and women of reproductive age [5].

In 2020, the World Health Organization (WHO) published new targets for STH control for 2030 [5]. The targets include the elimination of morbidity caused by STHs in SAC, defined as <2% moderate- and heavy-intensity (M&HI) infections, and a reduction in the number of anthelminthic tablets required for PC. As of 2017, 7 countries have achieved morbidity control, and 61 countries have achieved the minimum recommended PC coverage of 75% in pSAC and SAC [5]. Going forward to 2030, there will be an increased focus on sustaining achieved targets through tailored surveillance and interventions [5].

The WHO guidelines for STH control programs differentiate between low-, moderate- and high-prevalence settings, defined as <20%, 20–50%, and ≥50% prevalence of any STH infection in SAC, respectively [6]. The prevalence is typically determined by fecal smear tests. However, factors related to STH population biology and human behavior, for example, the distribution of worms among hosts and age-dependent exposure to infection, can affect the outcome of STH control programs and the meaning and implications of measuring certain prevalence levels. Although much has been achieved in STH control so far, these location-specific factors may become increasingly important to achieve sustainable STH control or elimination of transmission in the coming decade.

As an increasing number of countries report they are approaching the morbidity target (<2% M&HI infections in SAC), appropriate strategies need to be developed, employing sensitive diagnostics, to evaluate whether the target has been reached and to regularly monitor the prevalence of STH infections to detect signs of resurgence early on. Previous studies have shown that the accuracy of currently recommended sampling schemes and diagnostics used to measure STH prevalence is insufficient to assess achievement of the morbidity target [7]. The most commonly used STH diagnostics are based on egg counts from human stool samples, which have considerable measurement uncertainty [8, 9]. The number of eggs per gram feces (epg) is an indirect measure of infection intensity, and its interpretation in the context of STH control programs depends on assumptions about the relationship between egg counts and the number of fertilized female worms within a host. As this relationship is poorly understood, it contributes to the uncertainty in the evaluation of STH programs using models. At present, our knowledge depends on a few published worm expulsion studies, where the number of expelled worms are related to egg counts in stool samples [8, 9].

The purpose of this article is to review how uncertainty in key assumptions on STH population biology can affect the planning and evaluation of STH control programs and how this uncertainty can be accounted for in program design to achieve sustainable STH morbidity control. To illustrate this, we use 2 simulation models independently developed at Erasmus MC (EMC) and Imperial College London (ICL). The models have been described in detail elsewhere. The parameters used for the simulations can be found in Supplementary Table 1. The features of STH biology and human behavior that we consider here are the aggregation of worms within hosts (Supplementary Figure 1), the age-exposure profile of infection (Supplementary Figure 2), and the functional form of density-dependent fecundity of adult female worms.

## PROGRAM PLANNING

WHO guidelines for STH control programs recommend semi-annual PC treatment of pSAC and SAC in high-prevalence settings, annual PC treatment in moderate-prevalence settings, and no treatment in low-prevalence settings where M&HI infections are rare [6]. After 5–6 years, the guidelines recommend reevaluating the prevalence in SAC and adjusting PC frequency depending on the new prevalence value [6]. We discuss additional features of STH biology that affect the treatment frequency, duration, and coverage required to achieve the morbidity target.

### Treatment Frequency, Coverage, and Duration

The frequency and duration of treatment required to reach the morbidity target does not only depend on local prevalence but also on the aggregation of worms among hosts. Aggregation here means that most infected individuals will have low to moderate worm burdens, but a few individuals will have very high worm burdens. The negative binomial distribution has been proven to be a good descriptor of the distribution of worms per host. Typically, heavily infected individuals are predisposed to this state by high exposure and potentially host suitability for infection. Variation in exposure among individuals is caused by local differences in environmental variables and human behavior, which is expected to vary by cultural and socioeconomic setting [10, 11]. In settings with a high precontrol level of worm aggregation among hosts, the parasite population will be more resistant to extinction by PC treatment. In settings where PC is implemented, the level of aggregation may further increase because of imperfect PC coverage, and in particular in case of persistent noncompliance to treatment. Individuals with persistent high worm burdens due to lack of treatment or high risk of reinfection can sustain the environmental reservoir of infectious material and increase the risk of reinfection for the whole population.

**Figure 1.**
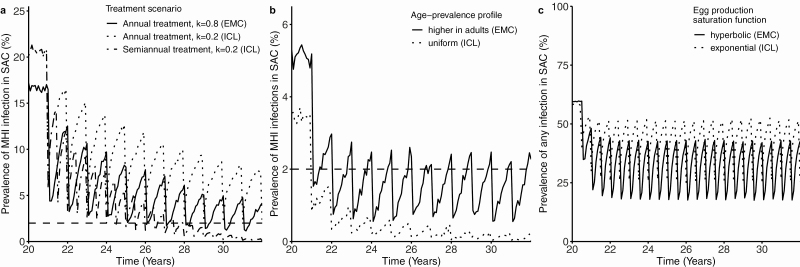
Impact of STH biology on the time course of control programs. Simulated scenarios have been aligned such that 95% of baseline prevalences of any infection of both models fall into the same range (20–50% for *Ascaris*, *a*, *b*; 50–70% for hookworm, *c*). Differences in the baseline prevalences of moderate-to-high intensity infections are due to different egg production functions used by the 2 models. (*a*) Example simulations for *A. lumbricoides*. Impact of aggregation on time to reaching the morbidity target (<2% moderate-to-high intensity infections, *dashed line*). Aggregation is inversely proportional to the parameter k. (*b*) Example simulations for hookworm. Impact of the age-prevalence profile on reaching the morbidity target. School-based treatment is less effective where the prevalence is higher in adults. (*c*) Example simulations for hookworm. Impact of density-dependent saturation of worm reproduction. In high prevalence settings, treatment is less impactful if exponential saturation of egg production is assumed. Abbreviations: EMC, Erasmus MC; ICL, Imperial College London; MHI, moderate and heavy intensity; STH, soil-transmitted helminth.

Locations with the same pretreatment prevalence of STH infections may have different levels of worm aggregation and may therefore require different optimal treatment strategies. In areas with a high aggregation of worms among hosts, achieving the morbidity target may require more time or a higher treatment frequency [12]. Simulation models with the same baseline prevalence but different aggregation levels show that in moderate-prevalence settings with a high aggregation of worms among hosts semi-annual PC treatment may be required to reach the morbidity target by 2030, whereas settings with the same baseline prevalence and lower aggregation can reach the target with annual PC treatment (Figure 1a). The prevalence of infection is related to the degree of worm aggregation (measured inversely by the negative binomial parameter k) [13].

To facilitate the achievement of control targets, it is desirable to reduce the level of aggregation of worms among hosts. Mathematical models predict that aggregation increases during treatment (Supplementary Figure 1). The reason is that treated individuals have zero or only a few worms, whereas untreated individuals are left with much higher worm burdens. Systematic noncompliance or nonaccess to treatment for which evidence is emerging in areas that have experienced many rounds of MDA can further increase post-treatment aggregation and make reaching the target more difficult [14–16]. Program planners should identify neglected populations and reasons why treatment has not been received as part of a targeted leave no one behind (LNOB) strategy. Aggregation can be reduced if exposure to infection in high-risk individuals is mitigated by targeted WASH interventions [17].

### Population Treated

Exposure of different age groups to STH infection can vary geographically depending on STH species and cultural and socioeconomic differences in human behavior. Differences in exposure can lead to differences in infection intensities. For example, the main burden of *A. lumbricoides* and *T. trichiura* infections lies in children, whereas hookworm infections affect adults equally or more than children [18–20]. The reason is that *A. lumbricoides* and *T. trichiura* are transmitted exclusively via the oral-fecal route, whereas hookworm larvae can infect humans directly via the skin [21]. Models can simulate differences in exposure by implementing exposure functions that result in observed infection intensities (Supplementary Figure 2). Models calibrated in this way predict that for STH species and settings where children experience the highest exposure to infection, school-based treatment is more effective than for species and settings where adults have a higher risk of exposure to infection (Figure 1b). In settings where hookworm is the dominant STH and the main burden of infection lies in adults, treating adults in addition to children may be necessary to control STH morbidity [14, 22], and adult treatment has the potential to speed up achievement of the WHO 2030 targets [23].

Age-dependent contribution to the environmental reservoir of infectious material (ie, defecation practices) can also impact on a program’s success. For example, in settings where adults contribute to environmental contamination similarly or more than children, PC treatment of adults is predicted to have greater additional benefits toward reaching the morbidity target than in settings where adult contamination of the environment is relatively low [24]. Extending WASH measures to the community, rather than focusing only on schools, will be especially important in these settings [25]. Thus, knowledge of local hygiene practices can inform decisions on extending treatment to adults.

## PROGRAM EVALUATION AND POST-PROGRAM SURVEILLANCE

Uncertainty about STH reproduction can affect program evaluation based on egg counts from fecal smear tests. Current WHO guidelines recommend evaluation of the impact of STH control programs after 5–6 years of PC treatment [6]. Important parasitological impact indicators include the prevalence of any STH infection and the prevalence of M&HI STH infections [26]. Sampling strategies recommend testing of SAC in sentinel sites from different ecological zones within a country, but the number of sentinel villages and SAC to be sampled and diagnostic tools have not been specified, and they are the subject of ongoing research [27]. Previous simulation studies have shown that the optimal number of sentinel villages depends on the heterogeneity of the prevalence distribution within an implementation unit and that sampling too few sentinel villages poses the risk of underestimating the prevalence of M&HI infections [28]. This may be particularly problematic if undersampled areas also have the least access to treatment.

The ability of an evaluation strategy to accurately assess the impact of STH control programs does not only depend on the sampling scheme but also on the diagnostic. Comparative experimental studies concluded that the sensitivity of the Kato-Katz fecal smear test is superior to other diagnostic methods based on egg counts [29]. However, the sensitivity of the Kato-Katz diagnostic varies by STH species, and the number of microscopy slides used and may be insufficient to inform decisions on stopping treatment once very low STH prevalences have been reached [30].

In addition to sampling and measurement uncertainty, there is an inherent uncertainty in the interpretation of diagnostic results for program evaluation because our understanding of how the number of eggs depends on the number of female worms within a host is incomplete.

### Evaluating Whether the Target Has Been Achieved

Programme evaluation is based on egg counts from human stool samples. The fecundity of female worms is affected by density-dependent competition for resources within the human host [31, 32]. When the number of worms within a host is small, density-dependent competition does not play a role, and the number of eggs produced increases linearly with the number of (fertilized) female worms. As the number of worms increases, the number of eggs produced per female worm decreases. The functional relationship between the number of worms within a host and the number of eggs produced per female worm is unknown.

Two different functions can accurately describe the density-dependent saturation of egg output per female worm. An exponential saturation function assumes that the total number of eggs produced by the worm population within a host first increases and then decreases as the number of female worms increases. A hyperbolic saturation function assumes that the total number of eggs produced within a host increases up to a threshold value as the number of female worms increases and stays stable thereafter.

**Figure 2.**
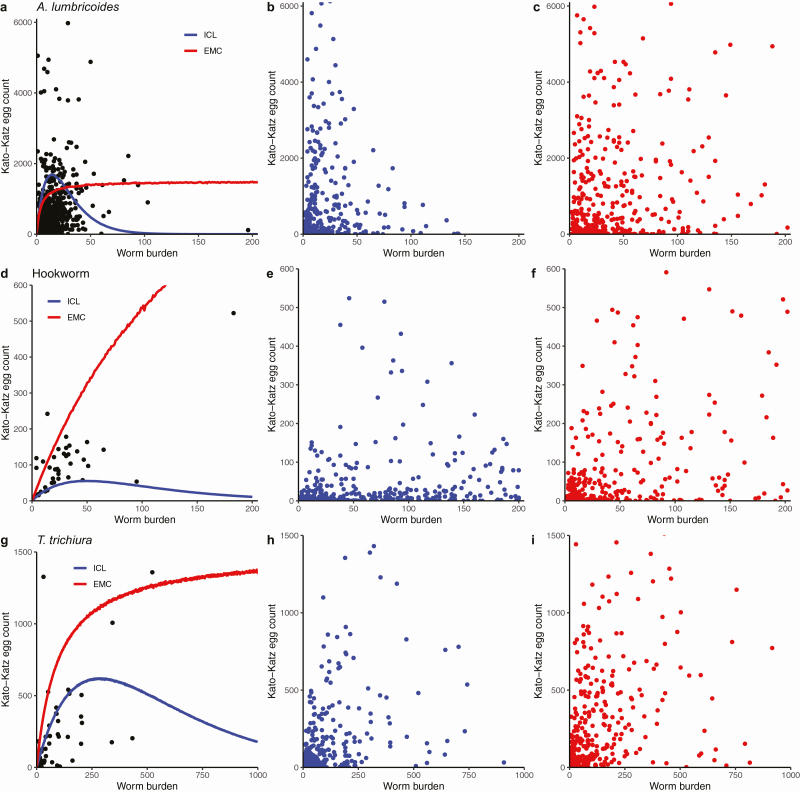
Individual-level egg count over individual-level worm burden data and simulations from EMC and ICL models. From top to bottom: *Ascaris*, hookworm, *Trichuris*. From left to right: real-world data (*A. lumbricoides* from Holland Asaolu 1991 [33], hookworm from Ghadirian 1979 [34], *Trichuris* from Bundy 1987 [20]), ICL model, EMC model. In column 1 the blue and red lines are the mean of 100 000 samples from the simulated egg production functions of the ICL and EMC models, respectively. Columns 2 and 3 show simulated data of one village of 500 individuals at a high equilibrium prevalence. Abbreviations: EMC, Erasmus MC; ICL, Imperial College London.

The choice of saturation function in mathematical models affects the predicted impact of PC treatment. For example, the impact of treatment on the prevalence of M&HI infections in high-prevalence settings is higher if the fecundity of female worms follows a hyperbolic saturation function. If the fecundity of female worms follows an exponential saturation function, the impact of treatment may be underestimated, because the number of worms within hosts may have been more reduced than the prevalence of M&HI infections measured by egg counts suggests (Figure 1c). The reason is that the egg count obtained from stool samples of hosts with very high worm burdens can be lower than in hosts with moderate worm burdens for exponential saturation of egg production. However, by looking at the available data from worm expulsion studies alone, we cannot decide which of the functions fits the data better, as this would require more worm expulsion data from highly infected individuals (Figure 2).

In moderate-prevalence settings, the impact of treatment on infection intensity depends less on the type of density-dependence and more on the slope of the egg production function. Consequently, the interpretation of evaluation data in moderate-prevalence settings is more straightforward than in high-prevalence settings.

Density-dependent fecundity of female STH can complicate the assessment of drug resistance using assays based on egg counts and may lead to an underestimation of drug efficacy. Veterinary studies in dogs have shown that dogs infected with high burdens of low-level resistant *Ancylostoma caninum* have higher egg outputs 6 days after deworming treatment than before, although the drug removed worms with 71% efficacy [35, 36]. The authors suggest that drug resistance should not be assessed in individuals with high infection intensity or that alternative assays should be used to assess suspected drug resistance—for example, in vitro assays observing the morphology or motility of STH larvae.

During program evaluation, a lower than expected reduction in STH prevalence and infection intensity in settings with a high baseline prevalence does not necessarily imply that the program was ineffective. However, efforts should be made to uphold high coverage and compliance until measurable progress has been achieved. More research is needed to establish the exact relationship between egg production and the number of female worms within a host. Data could be collected, for example, from worm expulsion studies from small cohorts at the beginning and end of STH control programs or from animal studies where model organisms are infected with known numbers of eggs/larvae.

### Avoiding Resurgence of STH Infections

Cessation of treatment or reduction in treatment frequency may lead to resurgence, unless elimination of transmission has been achieved or additional WASH interventions have been successfully implemented to reduce the value of the reproductive number R_0_ [37]. Careful post-program surveillance of STH infections is necessary to react quickly to possible resurgence [38]. The time to resurgence depends on the worm life expectancy and how close the post-treatment prevalence is to the transmission breakpoint [39].

Since the prevalence of STH infections and treatment coverage tend to be heterogeneous across a country, control targets are reached sooner in some implementation units than in others. To achieve targets and avoid resurgence in high-risk populations (eg, difficult-to-reach populations, children who do not attend school, populations with low MDA coverage, or poor compliance to repeated rounds of treatment, etc.), targeted setting-specific intervention strategies will have to be implemented. Such strategies need to consider the potential re-importation of STH infections by individuals moving between implementation units or across country borders, for example, seasonal migrant laborers [40].

## Summary

The current treatment recommendations for STHs are general and differentiate only by settings defined by the prevalence of infection. Considering local variability in epidemiologically relevant human behavior and STH population biology can improve STH control. Such considerations will become especially relevant to implement sustainable STH morbidity control once the current targets have been reached. Uncertainty about program design caused by uncertainty on STH biology, ecology, and human behavior can be addressed by collecting setting-specific data before implementing the full program, for example, collecting infection intensity data to estimate the level of aggregation of worms among hosts, collecting worm expulsion data at the beginning and at the end of MDA programs, especially, in high-prevalence settings (Table 1).

**Table 1.** Different Phases of STH Control Programs, How They Are Affected by Uncertainties Regarding STH Population Biology and Human Behaviour, and Recommendations to Reduce Uncertainty

Phase of STH Control Program	Uncertainty in STH Biology and Human Behaviour	Recommendation
Planning	Aggregation of worms among hosts	Collect infection intensity data in different age groups (pSAC, SAC, adults)
		Increase treatment frequency and/or coverage if the prevalence of M&HI is high
		Implement WASH and targeted Leave No One Behind strategies
	Age-dependent exposure to infection	Consider PC treatment for adults in addition to children where hookworm is the dominant STH species and prevalence or aggregation are high
	Age-dependent contribution to infection	Collect data on hygiene and defecation practices
		Consider PC treatment for adults in addition to children where the contribution to infection of adults is high and the impact of school-based treatment was less than expected
Monitoring and Evaluation	Density-dependent fecundity of worm reproduction	In high-prevalence settings a lower than expected programme impact on parasitological indicators may not mean that the programme was ineffective
		Consider collecting worm expulsion data at the start and during evaluatio n of the programme (especially in high-endemicity settings)

Abbreviations: M&HI, moderate- and heavy-intensity; PC, preventive chemotherapy; pSAC, preschool-age children; SAC, school-age children; STH, soil-transmitted helminth; WASH, water, sanitation, and hygiene.

Data that can inform the targeted design of STH control programs include the prevalence and intensity of STH infections by age group. To address uncertainty that cannot be reduced by collecting additional data prior to the implementation of STH control programs, contingency plans need to be made that account for local goals and resource availability and the consequences to future resource needs if program targets are not reached.

## Supplementary Data

Supplementary materials are available at *Clinical Infectious Diseases* online. Consisting of data provided by the authors to benefit the reader, the posted materials are not copyedited and are the sole responsibility of the authors, so questions or comments should be addressed to the corresponding author.

ciab195_suppl_Supplementary-Figures

ciab195_suppl_Supplementary-Table

## Notes

***Acknowledgments.*** The authors would like to thank James Truscott and Robert Hardwick for helpful discussions on the contents of the manuscript.

***Disclaimer.*** The views, opinions, assumptions, or any other information set out in this article are solely those of the authors and should not be attributed to the funders or any other person connected with the funders.

***Supplement sponsorship.*** This article appears as part of the supplement “Sustainable control of neglected tropical diseases – surveillance and diagnostics”, sponsored by the NTD Modelling Consortium.

***Financial support.*** This work was supported by the Bill and Melinda Gates Foundation the NTD Modelling Consortium (grant number OPP1184344). R. M. A. acknowledges funding via the DeWorm3 award by the Bill and Melinda Gates Foundation (grant number OPP1129535) to the Natural History Museum in London. LEC further acknowledges funding from the Dutch Society for Scientific Research (NWO, grant number 016.Veni.178.023). F. G. acknowledges funding from a European Marie Skłodowska-Curie fellowship (grant number H2020-COFUND-2015-FP-707404). The funders had no role in study design, data collection and analysis, decision to publish, or preparation of the manuscript.

***Potential conflicts of interest.*** R. A. reports share holding from GlaxoSmithKline, outside the submitted work. All other authors report no potential conflicts. All authors have submitted the ICMJE Form for Disclosure of Potential Conflicts of Interest. Conflicts that the editors consider relevant to the content of the manuscript have been disclosed.
