# Supplementary material for: Impact of Key Assumptions About the Population Biology of Soil-Transmitted Helminths on the Sustainable Control of Morbidity
Source: Clin Infect Dis. 2021 Jun 14;72(Suppl 3):S188–94. doi: 10.1093/cid/ciab195 (PMC8218855; doi:10.1093/cid/ciab195)

**Supplementary Information to “Impact of key assumptions about the population biology of soil-transmitted helminths on the sustainable control of morbidity”**

**Supplementary Figure 1**: Changes in k, the parameter describing the aggregation of worms among hosts, in SAC at three time points before and following the start of a PC programme for all three STH species, moderate prevalence settings. The aggregation parameter k was estimated before the subsequent treatment round. The x-axis gives the time from the start of the PC programme in years. Blue: ICL model. Red: EMC model.


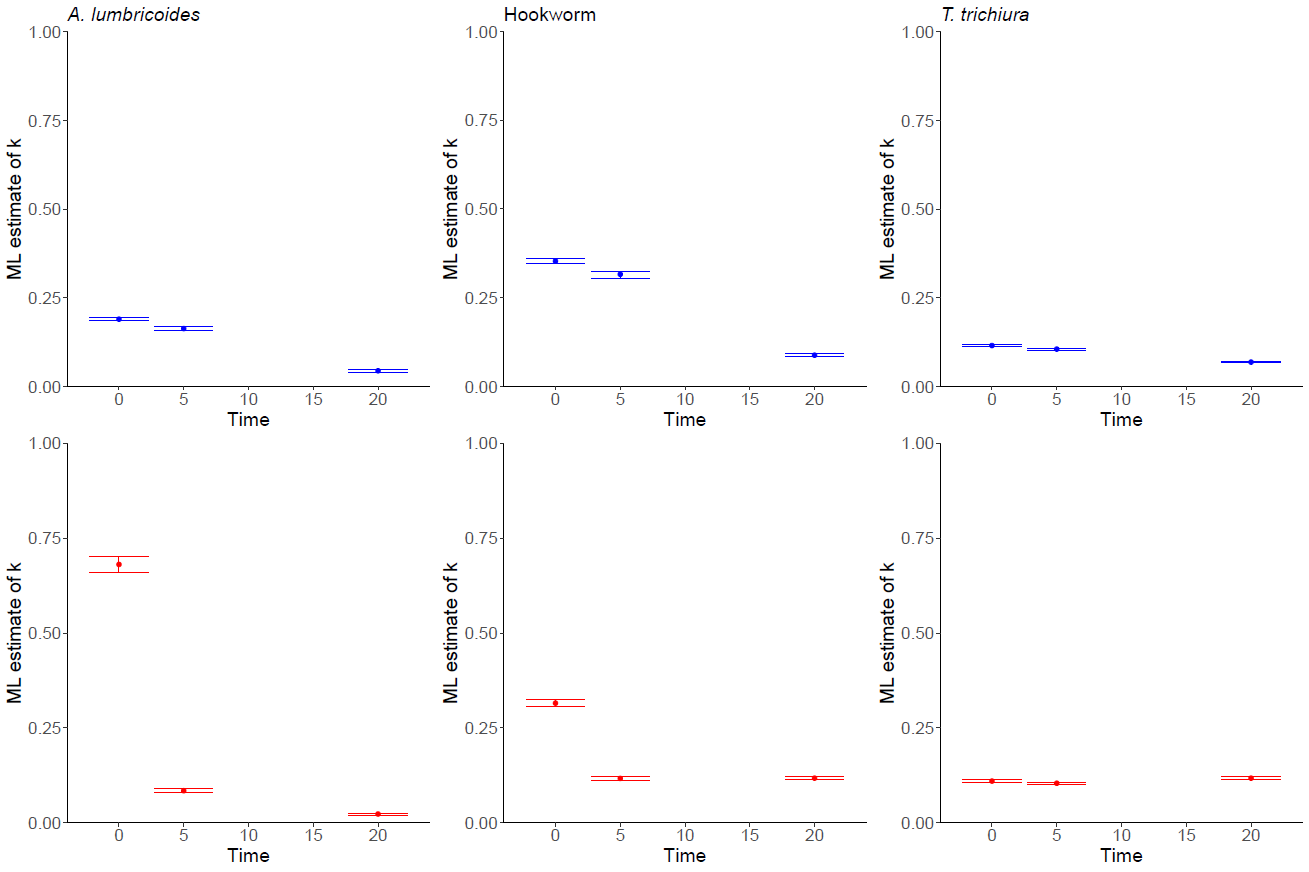


**Supplementary Figure 2**: Age-exposure and age-contribution functions for EMC (red) and ICL (blue) models for all three STH species.


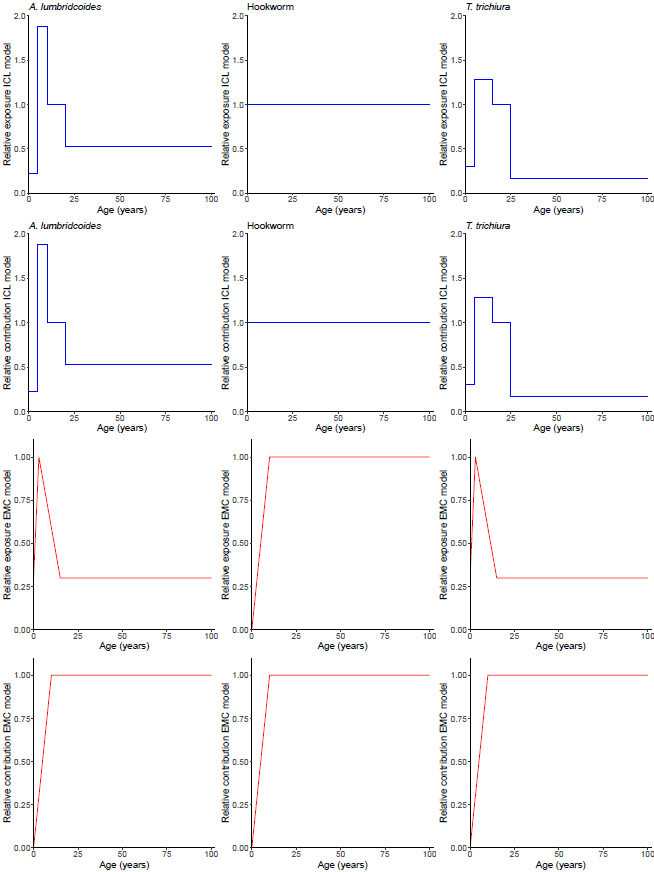

Supplement: ciab195_suppl_Supplementary-Figures [file ciab195_suppl_Supplementary-Figures.docx]
