# Supplementary material for: Impact of Key Assumptions About the Population Biology of Soil-Transmitted Helminths on the Sustainable Control of Morbidity
Source: Clin Infect Dis. 2021 Jun 14;72(Suppl 3):S188–94. doi: 10.1093/cid/ciab195 (PMC8218855; doi:10.1093/cid/ciab195)
Supplement: ciab195_suppl_Supplementary-Table [file ciab195_suppl_Supplementary-Table.docx]

Table S1. Model parameters used to simulate transmission of *Ascaris lumbricoides*, *Trichuris trichiura* and hookworm infections.

|  | **Value or assumption** | |
| --- | --- | --- |
| **Parameter** | **Erasmus MC** | **Imperial College London** |
| **Human demography** | | |
| *Hookworm* | Demographic data quantified for sub-Saharan Africa 2000 United Nations Population Division.^1^ | Demographic data taken from 2003 Kenya Demographic and Health Surveys.^2^ |
| *Ascariasis* | Demographic data quantified for sub-Saharan Africa 2000 United Nations Population Division.^1^ | Demographic data taken from 2003 Kenya Demographic and Health Surveys.^2^ |
| *Trichuris* | Demographic data quantified for sub-Saharan Africa 2000 United Nations Population Division.^1^ | Demographic data taken from 2003 Kenya Demographic and Health Surveys.^2^ |
| **Transmission of infection** | | |
| Aggregation of parasites in hosts |  |  |
| *Hookworm* | $k_{w}=0.35$ ^2^ | $k_{w}=0.35$ ^2^. |
| *Ascariasis* | $k_{w}=0.8$ ^3^ | $k_{w}=0.8$ ^3^,^4^ in high-prevalence settings,  $k_{w}=0.2$ in moderate-prevalence settings. |
| *Trichuris* | $k_{w}=0.38$, in high-prevalence settings fitted to data^5^,  $k_{w}=0.12$ in moderate-prevalence settings. | $k_{w}=0.38$, in high-prevalence settings fitted to data^5^,  $k_{w}=0.12$ in moderate-prevalence settings. |
| Variation in exposure and contribution to the environmental reservoir by age and sex |  |  |
| *Hookworm* | Relative exposure and contribution to the reservoir both increase linearly from 0 to 1 between ages 0–10 and is stable thereafter with no difference between males and females.^6^ | Relative exposure and contribution to the reservoir are assumed to vary be constant across age groups, assuming no difference between males and females. The values were estimated from baseline data of the Tumikia study^7^ and unpublished epidemiological data of the DeWorm3 study. |
| *Ascariasis* | Contribution to the reservoir increases linearly from 0 to 1 between ages 0–10 and is stable thereafter with no difference between males and females (reflecting behaviour related to defaecation and mobility patterns as previously estimated for hookworm^6^). Exposure to the reservoir is defined as a piece-wise linear function of age that increases linearly from a base level $x_{0}$ =0.33 of relative exposure at age zero to a relative exposure of 1.0 at age $a_{\text{peak}}$=3, and then again linearly declines back to the base level $x_{0}$ at age 15 and is stable thereafter. This function aims to reflect behaviour leading to ingestion of contaminated matter, which typically peaks in young children.^3^ | Relative exposure and contribution to the reservoir by age are assumed to be equal and are estimated from^3^ : 0.22 (0-4 years), 1.88 (5-9), 1.0 (10-19), 0.53 (20+). |
| *Trichuris* | Contribution to the reservoir increases linearly from 0 to 1 between ages 0–10 and is stable thereafter with no difference between males and females (reflecting behaviour related to defaecation and mobility patterns as previously estimated for hookworm^6^). Exposure to the reservoir is defined as a piece-wise linear function of age that increases linearly from a base level $x_{0}$ =0.33 of relative exposure at age zero to a relative exposure of 1.0 at age $a_{\text{peak}}$=3, and then again linearly declines back to the base level $x_{0}$ at age 15 and is stable thereafter. This function aims to reflect behaviour leading to ingestion of contaminated matter, which typically peaks in young children.^3^ | Relative exposure and contribution to the reservoir are assumed to vary piece-wise constant by age group and are estimated at 0.3 (0-4 years), 1.28 (5-14), 1 (15-24) and 0.17 (ages 25+), assuming no difference between males and females. These figures were estimated from epidemiological data.^5^ |
| **Life history and productivity of the parasite in the human host** | | |
| Average worm lifespan |  |  |
| *Hookworm* | 3 years ^8-10^ | 2 years ^11^ |
| *Ascariasis* | 1 year ^3^,^8-10^,^12^ | 1 year ^8-10^,^12^ |
| *Trichuris* | 1 year ^5^,^11^ | 1 year ^5^,^11^ |
| Variation in worm lifespan | Weibull distribution with shape 2; i.e. the mortality rate is zero at age zero and then increases linearly with worm age (assumption as previously used for hookworm ^6^). | Exponential distribution; i.e. the mortality rate is constant and independent of worm age. |
| Pre-patent period |  |  |
| *Hookworm* | 7 weeks ^8^,^9^,^13^,^14^ | No pre-patent period used. |
| *Ascariasis* | 10 weeks ^8^ | No pre-patent period used. |
| *Trichuris* | 10 weeks ^8^ | No pre-patent period used. |
| Age-dependent reproductive capacity | Constant over age (assumption). | Constant over age (assumption). |
| Mode of reproduction | Polygamous sexual reproduction. The probability of a fertilised egg to develop into a male or a female worm follows a binomial distribution with p=0.5. | Polygamous sexual reproduction. The probability of a fertilised egg to develop into a male or a female worm follows a binomial distribution with p=0.5. |
| Female worm fecundity | Density-dependent on total number of female worms in host, assuming hyperbolic saturation.^6^ | Density-dependent on total number of female worms in host, assuming exponential saturation. Exponential model of saturation with parameter γ = 0.02 ^15^ for hookworm, γ = 0.07 for ascaris ^4^ and γ = 0.0035.^5^,^16^ |
| *Hookworm* | On average 8.3 eggs per female worm per 41.7 mg sample of faeces (200 epg per female worm, as previously reported based on association between number of expulsed adult female worms and egg counts based on Kato-Katz^17^). The average maximum total host output is assumed to be 62.5 eggs per 41.7 mg faeces (1500 epg, as previously assumed^6^). | On average 3 eggs per female worm per 41.7 mg sample of faeces (72 epg per female worm, as previously reported based on association between number of expulsed adult female worms and egg counts based on Kato-Katz^17^). |
| *Ascariasis* | On average 406 eggs per female worm per 41.7 mg sample of faeces (9750 epg per female worm), and maximum total host output of 777 eggs per 41.7 mg faeces on average (18,650 epg). These figures were estimated from pre-control data on number of expulsed adult female worms and egg counts based on a concentration and sedimentation technique using homogenised stools.^3^ | On average 320 eggs per female worm per 41.7 mg sample of faeces (7674 epg per female worm). |
| *Trichuris* | On average 15.4 eggs per female worm per 41.7 mg sample of faeces (370 epg per female worm), and maximum total host output of 3333.33 eggs per 41.7 mg faeces on average (80,000 epg). These figures were estimated from pre-control data on number of expulsed adult female worms and egg counts based on a concentration and sedimentation technique using homogenised stools.^3^ | On average 5.875 eggs per female worm per 41.7 mg sample of faeces (141 epg per female worm).^5^ |
| Host immunity to incoming infections | None (assumption). | None (assumption). |
| **Infection dynamics in environmental reservoir** | | |
| Survival of infective material in the central reservoir | Exponential survival (assumption). | Exponential survival (assumption). |
| *Hookworm* | Average lifespan of two weeks, implemented as a monthly survival probability of $\exp\left( -26/12 \right)=11.5\%$ (95%-CI: 0.05–7.38 weeks under assumption of exponential survival), based on the notion that average survival time is in the order of weeks.^13^,^14^,^18^ | Average lifespan of 30 days.^11^ |
| *Ascariasis* | Average lifespan of 1.5 month, implemented as a monthly survival probability of $\exp\left( -1/1.5 \right)=51.3\%$ (95%-CI: 0.04–5.53 months under assumption of exponential survival).^9^,^10^ | Average lifespan 2 months.^19^ |
| *Trichuris* | Average lifespan of 20 days implemented as a monthly survival probability of $\exp\left( -1/(20/ 30 \right))=22.3\%$ (95%-CI: 0.02–2.46 months under assumption of exponential survival. | Average lifespan 20 days.^5^ |
| **Drug treatment** | | |
| Proportion of adult worms killed by single dose of albendazole (400 mg), or pyrantel pamoate (10 mg/kg, ascariasis only) | Assumption: proportion killed is equal to the faecal egg reduction rate. | Assumption: proportion killed is equal to the faecal egg reduction rate. |
| *Hookworm* | 0.95 for albendazole.^20^ | 0.95 for albendazole.^20^ |
| *Ascariasis* | 0.99 for albendazole.^20^ | 0.99 for albendazole.^20^ |
| *Trichuris* | 0.60 for albendazole.^20^ | 0.60 for albendazole.^20^ |
| Coverage | 75% coverage in pre-school-age children and school-age children (assumption) | 75% coverage in pre-school-age children and school-age children (assumption) |
| Compliance/access to treatment | Assumed to be random here | Assumed to be random here |
| **Diagnostic test outcomes** |  |  |
| Variability in measured host load of infective material (eggs per examined sample of faeces) |  |  |
| *Hookworm* | Kato-Katz: negative binomial distribution with aggregation parameter $k=0.35$, estimated separately from repeated individual-level egg count data from Uganda.^21^ | Kato-Katz: negative binomial distribution with aggregation parameter $k=0.35$, estimated from unpublished triple egg count data from Tamil Nadu, India |
| *Ascariasis* | Kato-Katz: negative binomial distribution with aggregation parameter $k=0.25$.^22^ | Kato-Katz: negative binomial distribution with aggregation parameter $k=0.3$ ^22^ |
| *Trichuris* | Kato-Katz: negative binomial distribution with aggregation parameter $k=0.82$.^16^ | Kato-Katz: negative binomial distribution with aggregation parameter $k=0.82$ ^16^ |

Other values used in model: Cut-offs for light moderate and heavy infection in eggs per gram stool, according to WHO guidelines.

|  |  |  |
| --- | --- | --- |
| *Hookworm* | 1, 2000, and 4000 epg | 1, 2000, and 4000 epg |
| *Ascariasis* | 1, 5000, and 50,000 epg | 1, 5000, and 50,000 epg |
| *Trichuris* | 1, 1000, and 10,000 epg | 1, 1000, and 10,000 epg |

**References**

1. Jambulingam P, Subramanian S, de Vlas SJ *et al.* Mathematical modelling of lymphatic filariasis elimination programmes in India: required duration of mass drug administration and post-treatment level of infection indicators. *Parasit Vectors* 2016; 9:501.

2. Bradley M, Chandiwana SK, Bundy DAP *et al.* The epidemiology and population biology of Necator americanus infection in a rural community in Zimbabwe. *Trans R Soc Trop Med Hyg* 1992; 86:73–6.

3. Elkins DB, Haswell-Elkins M, Anderson RM. The epidemiology and control of intestinal helminths in the Pulicat Lake region of Southern India. I. Study design and pre- and post-treatment observations on Ascaris lumbricoides infection. *Trans R Soc Trop Med Hyg* 1986; 80:774–92.

4. Truscott JE, Turner HC, Farrell SH *et al.* Soil-Transmitted Helminths: Mathematical Models of Transmission, the Impact of Mass Drug Administration and Transmission Elimination Criteria. *Adv Parasitol* 2016; 94:133–98.

5. Bundy DA, Cooper ES, Thompson DE *et al.* Age-related prevalence and intensity of Trichuris trichiura infection in a St. Lucian community. *Trans R Soc Trop Med Hyg* 1987; 81:85–94.

6. Coffeng LE, Bakker R, Montresor A *et al.* Feasibility of controlling hookworm infection through preventive chemotherapy: a simulation study using the individual-based WORMSIM modelling framework. *Parasit Vectors* 2015; 8:541.

7. Truscott JE, Ower AK, Werkman M *et al.* Heterogeneity in transmission parameters of hookworm infection within the baseline data from the TUMIKIA study in Kenya. *Parasit Vectors* 2019; 12:442.

8. Bethony J, Brooker S, Albonico M *et al.* Soil-transmitted helminth infections: ascariasis, trichuriasis, and hookworm. *Lancet* 2006; 367:1521–32.

9. Anderson RM, Truscott J, Hollingsworth TD. The coverage and frequency of mass drug administration required to eliminate persistent transmission of soil-transmitted helminths. *Philos Trans R Soc Lond B Biol Sci* 2014; 369:20130435.

10. Truscott JE, Hollingsworth TD, Brooker SJ *et al.* Can chemotherapy alone eliminate the transmission of soil transmitted helminths? *Parasit Vectors* 2014; 7:266.

11. Anderson RM, May RM. Helminth infections of humans: mathematical models, population dynamics, and control. *Adv Parasitol* 1985; 24:1–101.

12. Croll NA, Anderson RM, Gyorkos TW *et al.* The population biology and control of Ascaris lumbricoides in a rural community in Iran. *Trans R Soc Trop Med Hyg* 1982; 76:187–97.

13. Hotez PJ, Brooker S, Bethony JM *et al.* Hookworm infection. *N Engl J Med* 2004; 351:799–807.

14. Brooker S, Bethony J, Hotez PJ. Human Hookworm Infection in the 21st Century. *Adv Parasitol* 2004, 58:197–288.

15. Coffeng LE, Truscott JE, Farrell SH *et al.* Comparison and validation of two mathematical models for the impact of mass drug administration on Ascaris lumbricoides and hookworm infection. *Epidemics* 2017; 18:38–47.

16. Turner HC, Truscott JE, Bettis AA *et al.* Analysis of the population-level impact of co-administering ivermectin with albendazole or mebendazole for the control and elimination of Trichuris trichiura. *Parasite Epidemiol Control* 2016; 1:177–87.

17. Anderson RM, Schad GA. Hookworm burdens and faecal egg counts: an analysis of the biological basis of variation. *Trans R Soc Trop Med Hyg* 1985; 79:812–25.

18. Augustine DL. Investigations on the control of hookworm disease. XVI. Length of life of hookworm larvae from the stools of different individuals. *Am J Epidemiol* 1923; 3:127–36.

19. Anderson RM, May RM. Population dynamics of human helminth infections: control by chemotherapy. *Nature* 1982; 297:557–63.

20. Levecke B, Montresor A, Albonico M *et al.* Assessment of anthelmintic efficacy of mebendazole in school children in six countries where soil-transmitted helminths are endemic. Olliaro PL (ed.). *PLoS Negl Trop Dis* 2014; 8:e3204.

21. Pullan RL, Kabatereine NB, Quinnell RJ *et al.* Spatial and Genetic Epidemiology of Hookworm in a Rural Community in Uganda. Bethony JM (ed.). *PLoS Negl Trop Dis* 2010; 4:e713.

22. Easton AV, Oliveira RG, Walker M *et al.* Sources of variability in the measurement of Ascaris lumbricoides infection intensity by Kato-Katz and qPCR. *Parasit Vectors* 2017; 10:256.
